# Egg consumption and cardiovascular risk: a dose–response meta-analysis of prospective cohort studies

**DOI:** 10.1007/s00394-020-02345-7

**Published:** 2020-08-31

**Authors:** Justyna Godos, Agnieszka Micek, Tomasz Brzostek, Estefania Toledo, Licia Iacoviello, Arne Astrup, Oscar H. Franco, Fabio Galvano, Miguel A. Martinez-Gonzalez, Giuseppe Grosso

**Affiliations:** 1 Oasi Research Institute, IRCCS, Troina, Italy; 2 Department of Nursing Management and Epidemiology Nursing, Faculty of Health Sciences, Jagiellonian University Medical College, Kraków, Poland; 3 Department of Internal Medicine and Community Nursing, Faculty of Health Sciences, Jagiellonian University Medical College, Kraków, Poland; 4 Department of Preventive Medicine and Public Health, School of Medicine, University of Navarra, Pamplona, Navarre Spain; 5 CIBER Physiopathology of Obesity and Nutrition (CIBEROBN), Carlos III Institute of Health, Madrid, Spain; 6 Navarra Institute for Health Research, IdiSNA, Pamplona, Navarre Spain; 7 Department of Epidemiology and Prevention, IRCCS NEUROMED, Pozzilli, IS Italy; 8 Department of Medicine and Surgery, Research Centre in Epidemiology and Preventive Medicine (EPIMED), University of Insubria, Varese, Italy; 9 Department of Nutrition, Exercise, and Sports, Faculty of Science, University of Copenhagen, Nørre Campus, Copenhagen, Denmark; 10 Department of Epidemiology, Erasmus MC, University Medical Center Rotterdam, Rotterdam, Netherlands; 11 Institute of Social and Preventive Medicine, University of Bern, Bern, Switzerland; 12 Department of Biomedical and Biotechnological Sciences, University of Catania, Via S. Sofia 97, 95123 Catania, Italy; 13 Department of Nutrition, Harvard T.H. Chan School of Public Health, Boston, MA USA

**Keywords:** Egg, Cardiovascular disease, Stroke, Prospective cohort, Meta-analysis, Dose–response

## Abstract

**Purpose:**

Cardiovascular disease (CVD) is a leading cause of mortality globally and is strongly influenced by dietary risk factors. The aim was to assess the association between egg consumption and risk of CVD risk/mortality, including coronary heart disease (CHD), stroke, and heart failure.

**Methods:**

MEDLINE, Embase, and Web of Science databases were searched through April 2020 for prospective studies. Two independent reviewers screened and extracted the data through standardized methods. Size effects were calculated as summary relative risks (SRRs) in a dose–response fashion through random-effects meta-analyses.

**Results:**

Thirty-nine studies including nearly 2 million individuals and 85,053 CHD, 25,103 stroke, 7536 heart failure, and 147,124 CVD cases were included. The summary analysis including 17 datasets from 14 studies conducted on CVD (incidence and/or mortality) showed that intake of up to six eggs per week is inversely associated with CVD events, when compared to no consumption [for four eggs per week, SRR = 0.95 (95% CI: 0.90; 1.00)]; a decreased risk of CVD incidence was observed for consumption of up to one egg per day [SRR = 0.94 (95% CI: 0.89; 0.99)]. The summary analysis for CHD incidence/mortality including 24 datasets from 16 studies showed a decreased risk up to two eggs per week [(SRR = 0.96 (95% CI: 0.91; 1.00)]. No associations were retrieved with risk of stroke. The summary analysis for heart failure risk including six datasets from four studies showed that intake of one egg per day was associated with increased risk raising for higher intakes compared to no consumption [for 1 egg per day, SRR = 1.15 (95% CI:1.02; 1.30)]. After considering GRADE criteria for strength of the evidence, it was rated low for all outcomes but stroke, for which it was moderate (yet referring to no risk).

**Conclusion:**

There is no conclusive evidence on the role of egg in CVD risk, despite the fact that higher quality studies are warranted to obtain stronger evidence for a possible protection of CVD associated with moderate weekly egg consumption compared to no intake; equally, future studies may strengthen the evidence for increased heart failure risk associated with high regular egg consumption.

**Electronic supplementary material:**

The online version of this article (10.1007/s00394-020-02345-7) contains supplementary material, which is available to authorized users.

## Introduction

Cardiovascular disease (CVD) represents the leading cause of mortality globally, responsible for a total of about 18 million deaths in 2017, while increasing from 12.3 million in 1990 [1]. Nutritional risk factors have been considered of paramount importance to prevent the global burden of CVD [2,3]. Among the many factors widely studied over the last decades, dietary cholesterol has been the focus of major attention due to the relationship between blood cholesterol and increased risk of CVD firstly observed in the Framingham Heart Study nearly half century ago and ever since considered as risk factor [4]. Eggs, as major sources of dietary cholesterol (200–300 mg/100 g, about 180 mg per medium egg), have been subsequently advised to be consumed in moderation to lower dietary cholesterol intake [5]. However, current evidence on the association between dietary cholesterol and CVD risk is not consistent [6]. In 2000 the American Heart Association advised consumption of up to one egg per day [7] and nearly 10 years later the US Dietary Guidelines Advisory Committee eliminated cholesterol restrictions from the latest US dietary guidelines [8]. Nonetheless, the general opinion on egg consumption might be misled and food advertising and media campaigns sponsoring and claiming cholesterol-free products as healthier (sometimes supplemented with added sugars) are common. As specifically for egg consumption, a comprehensive summary of evidence reported repeatedly null and contrasting findings, suggesting that meta-analytic studies need to better investigate potential confounding effects of relevant variables (i.e., sex, geographical area, adjustment for health or dietary variables, etc.) [9]. However, more prospective cohort studies have been published so far: specifically, a later study involving 6 US cohorts showed that egg consumption was associated with increased risk of CVD and that the detrimental cardiovascular effect of egg consumption was mainly driven by dietary cholesterol, once more suggesting the need to limit eggs consumption. In light of such considerations, the aim of this study was to update current evidence on the association between egg consumption and CVD risk while assessing whether confounding factors may play a role in such relation.

## Methods

### Study design

The design, analysis, and reporting of this study followed the meta-analysis of Observational Studies in Epidemiology (MOOSE) guidelines (ESM Table 1). A systematic search on PubMed (https://www.ncbi.nlm.nih.gov/pubmed/), EMBASE (https://www.embase.com/), Web of Science (www.webofknowledge.com) databases of studies published up to April 2020 was performed with the following search strategy: “[(egg OR eggs) AND (coronary heart disease OR myocardial infarction OR ischemic heart disease OR ischemic heart disease OR coronary artery disease OR heart disease OR stroke OR cardiovascular disease OR heart failure)] AND (cohort OR prospective OR longitudinal OR follow-up)”. Studies were selected if they met the following inclusion criteria: (i) they were conducted on general population of human adults (i.e., no patients recruited in hospitals); (ii) had a prospective design; (iii) evaluated associations between egg intake and risk of CVD (fatal and non-fatal), cardiovascular-related outcomes (such as coronary heart disease [CHD] and stroke, fatal and non-fatal), and heart failure; (iv) assessed and reported hazard ratios (HRs) or risk ratios (RRs) and their corresponding 95% CI for ≥ 3 exposure categories (egg consumption) or provided HRs for increased intake of egg (as a continuous variable); and (v) provided a defined amount of egg consumption per category of exposure (i.e., servings of eggs per day or week). Reference lists of studies of interest were also examined for any additional study not previously identified. If more than one study was conducted on the same cohort, only the dataset including the larger number of individuals, the longest follow-up, or the most comprehensive data (i.e., number of cases and person-year for each category of exposure) was included on a case by case situation, depending on the analysis performed (see below). We did not exclude studies based on language or publication date. All references were evaluated by two independent reviewers (J.G., G.G.) with a third reviewer (A.M.) available in case of disagreement.

### Data extraction

Data were abstracted by the two independent reviewers from each identified study using a standardized extraction form. The following information was collected: (i) first author name; (ii) year of publication; (iii) study cohort name and country; (iv) number, sex, and age (mean or range) of participants; (v) follow-up period; (vi) endpoints and cases; (vii) distributions of cases and person-years, HRs and 95% CIs for all categories of exposure; (viii) covariates used in adjustments.

### Risk of bias and quality assessment

Risk of bias was assessed using the Cochrane Risk of bias in Non-randomized Studies of Interventions (ROBINS-I) tool previously used in comprehensive meta-analyses with similar outcomes [10, 11]. The tool consists of the following seven domains: (1) confounding, (2) selection of participants, (3) measurement of the exposure, (4) misclassification of exposure during follow-up, (5) missing data, (6) measurement of outcomes and (7) selective reporting. Two researchers (J.G. and A. M.) assessed the risk of bias independently. Any disagreements were resolved by consensus or by consultation of a third researcher.

### Outcomes

Outcomes evaluated in the analyses included total CVD, CHD, and stroke (including sub-types hemorrhagic and ischemic stroke) incidence and mortality. Also risk of heart failure incidence was assessed.

### Statistical analysis

When egg consumption was reported by ranges of intake, the midpoint of the range was used. When the highest category was open-ended, we assumed the width of the category to be the same as the adjacent category. When the lowest category was open-ended, we set the lower boundary to zero. Two-stage random-effects dose–response meta-analysis was performed to examine linear and non-linear relationship between egg consumption and CVD outcomes. In the first stage the method reported by Greenland and Orsini (generalized least-squares, GLS) was used to calculate study-specific coefficients on the basis of results across categories of egg consumption taking into account the correlation within each set of retrieved HRs [12,13]. Non-linear dose–response analysis was modeled using restricted cubic splines with three knots at fixed percentiles (25%, 50%, and 75%) of the distribution [14]. We combined the coefficients that had been estimated within each study by performing random-effects meta-analysis. In linear dose–response meta-analysis the method of DerSimonian and Laird was used and in non-linear dose–response meta-analysis the multivariate extension of the method of moments was used to estimate summary relative risks (SRRs). We calculated an overall *P *value by testing that the two regression coefficients were simultaneously equal to zero. We then calculated a *P *value for non-linearity by testing that the coefficient of the second spline was equal to zero. A subgroup analysis was conducted for those studies providing risk measures by diabetic status. A number of sensitivity analyses were conducted to test stability of results, including (i) exclusion of one study at the time, (ii) exclusion of studies that did not report number of cases and person-years for each category of exposure, and (iii) stratifying studies by variables of interest (such as sex, geographical localization of the cohort, level of adjustment for body mass index [BMI], diabetic status, and other dietary factors, and study quality). To facilitate interpretation of the results and easy application for dietary advices for the general population, the analyses were provided in depth for arbitrarily defined doses, such as “habitual” (daily) egg consumption corresponding to one egg per day, and “moderate” (weekly) egg consumption corresponding to four eggs per week. Publication bias was assessed with Egger’s regression test. Statistical heterogeneity between studies was assessed using the *χ*^2^ test (defined as a *P* value less than 0.10) and quantified through the multivariate generalization of the *I*^*2*^ statistic. All analyses were performed with R software version 3.0.3, dosresmeta and mvmeta packages (Development Core Team, Vienna, Austria).

### Grading of the evidence

The certainty of the evidence was assessed using the Grading of Recommendations, Assessment, Development, and Evaluation (GRADE) system [15]. Included observational studies started at low-certainty of evidence by default and then were downgraded or upgraded based on pre-specified criteria. Criteria to downgrade certainty included study limitations (weight of studies showing risk of bias by ROBINS-I), inconsistency (substantial unexplained inter-study heterogeneity, *I*^2^ ≥ 50% and *P*_het_ < 0.10), indirectness (presence of factors relating to the population, exposures and outcomes that limit generalizability), imprecision [95% CIs were wide or crossed a minimally important difference of 5% (SRR 0.95–1.05) for all CVD outcomes] and publication bias [significant evidence of small-study effects). Criteria to upgrade included a large effect size (SRR > 2 or SRR < 0.5 in the absence of plausible confounders], a dose–response gradient and attenuation by plausible confounding effects.

## Results

### Study characteristics

**Fig. 1:**
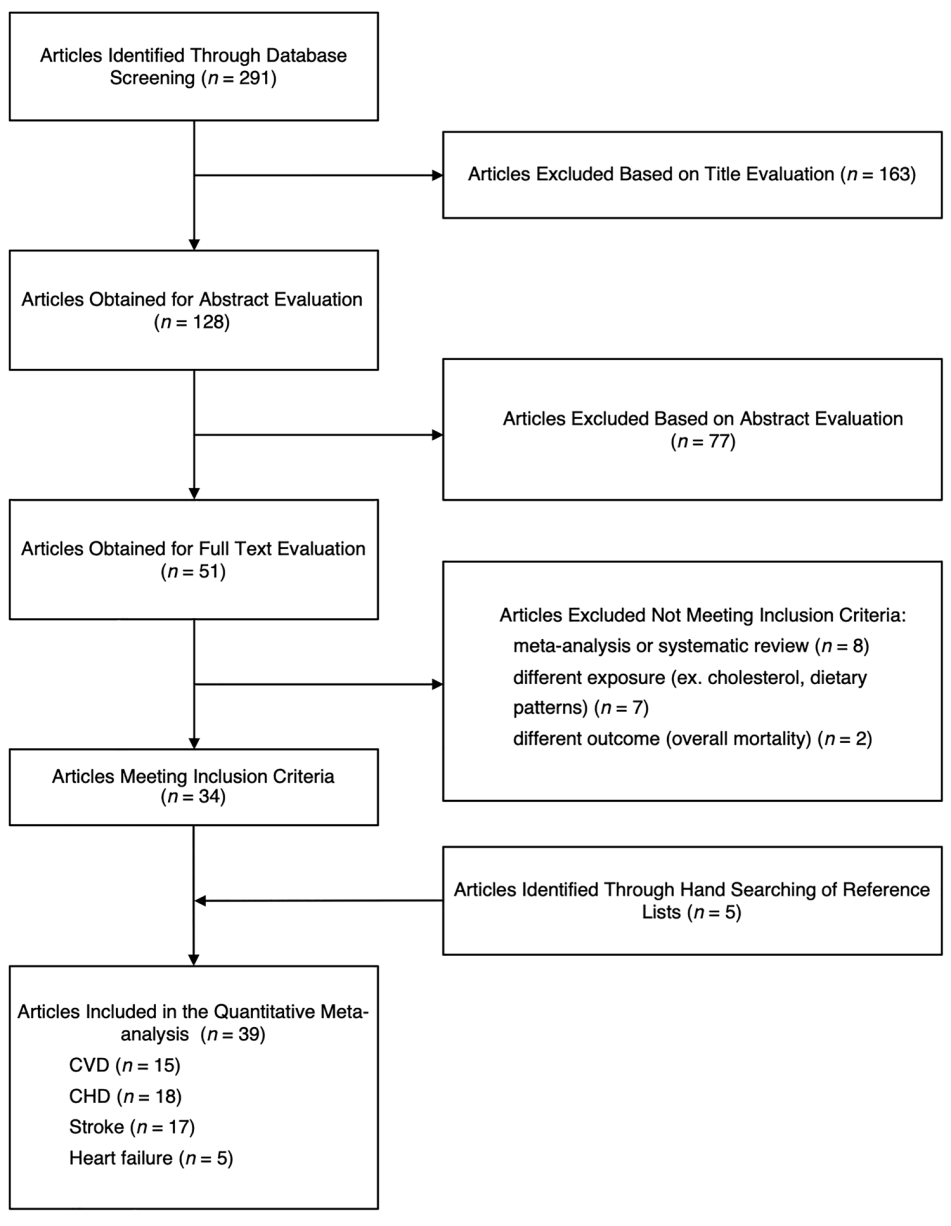
Flow chart of study identification and selection process

Out of 291 initial references identified, a total of 39 studies [16–54] were selected based on 38 cohorts providing data on CHD (1,831,038 individuals and 85,053 cases), stroke (761,962 individuals and 25,103 cases), heart failure (254,588 individuals and 7536 cases), and CVD (1,117,033 individuals and 147,124 cases) outcomes (Fig. 1). A detailed description of the studies included is presented in Table 1. From the 38 individual cohorts, 16 were from North America, 9 from Europe, 9 from Asia and one from Iran, and 3 multinational cohorts. One of the studies from North America included a pooled analysis of 6 US cohorts (pooled data was used in this meta-analysis). All studies had adequate follow-up to assess occurrence of the outcomes investigated (ranging from 3 to 32 years of mean follow-up). All studies scored moderate or serious risk of bias; a detailed description of judgment of potential risk of bias is given in the online supplementary materials (ESM Table 2). All but four studies [25, 28, 32, 34] provided full data of interest for better risk estimation (number of cases and person-years for each category of exposure), most of studies reported analyses adjusted for potential confounders investigated: among other dietary factors, besides total energy intake nearly always considered, also intake of other food groups (fruit/vegetable, whole grains, meat), macronutrients (trans-fats, protein) and fiber have been considered. Subgroup analyses were conducted through sex- and diabetic-specific groups, including nine studies provided separate risk estimates for male and female participants, and eight studies for diabetic participants.

**Table 1:** Characteristics of the prospective cohort studies selected for meta-analysis

References	Cohort name, years of study (country)	Follow-up	Sample, sex, age	Outcomes, no. of cases	Diet assessment	Covariate adjustment
Hu [16]	HPFS, 1986 and NHS, 1980 (US)	8 years in men and 14 years in women	37,851 men (40–75 years) and 80,082 women (34–59 years)	866 CHD and 258 stroke events in men and 939 CHD and 563 stroke events in women	Repeated FFQ	Age, BMI, 2-year history of myocardial infarction, multivitamin supplement use, vitamin E, menopausal hormone use (women), history of hypertension, physical activity, and total energy intake
He [17]	HPFS, 1986 (US)	14 years	43,732 (40–75 years) men	725 stroke, 455 ischemic stroke, 125 hemorrhagic stroke events	Repeated FFQ	BMI, physical activity, history of hypertension, smoking status, aspirin use, multivitamin use, consumption of alcohol, potassium, fiber, vitamin E, total servings of fruit and vegetables, total energy intake, and hypercholesterolemia at baseline
Sauvaget [18]	LSS, 1979–1981 (Japan)	16 years	15,350 men (mean age 54 years) and 24 999 women (mean age 58 years)	1462 stroke events	FFQ	Stratified by sex and birth cohort, adjusted for city, radiation dose, self-reported BMI, smoking status, alcohol habits, education level, history of diabetes, or hypertension
Nakamura [19]	NIPPON DATA80, 1980 (Japan)	14 years	5186 women (≥ 30 years) and 4077 men (≥ 30 years)	112 stroke and 39 CHD events in men, 107 stroke and 41 IHD events in women	FFQ	Age, serum creatinine, total cholesterol, blood glucose, BMI, systolic and diastolic blood pressures, use of blood pressure–lowering drugs, cigarette smoking, and alcohol intake
Nakamura [20]	JPHC, 1990 (Japan)	10.2 years (mean)	19,856 men and 21,408 women, aged 40–59 years in cohort I; 23,463 men and 26,008 women, aged 40–69 years in cohort II	462 CHD events	FFQ	Age, sex, BMI, hypertension, diabetes, use of cholesterol-lowering drugs, smoking, alcohol drinking, whether or not intended to avoid cholesterol-rich diets, consumption frequencies of meat, fish, vegetables, fruits, and cohort effects
Trichopoulou [52]	EPIC-Greece, 1994–1999 (Greece)	4.5 years (mean)	1013 men and women (20–86 years)	46 CVD death events	FFQ	Gender, age, educational level, smoking, waist-to-height, hip circumference, MET score, treatment with insulin, treatment for hypertension at enrollment, treatment for hypercholesterolemia at enrollment, and other indicated food groups
Qureshi [21]	NHANES I, 1982–1992 (US)	20 years	13,586 men and women (25–74 years)	655 stroke, 1584 MI and 253 CVD death events	FFQ	Age, gender, race/ethnicity, systolic blood pressure, diabetes mellitus, serum cholesterol, cigarette smoking, BMI, and educational status
Djoussé [22]	PHS, 1981 (US)	20 years	21,327 men (40–85 years)	1550 MI, 1342 stroke events	FFQ	Age, BMI, smoking, history of hypertension, vitamin intake, alcohol consumption, vegetable consumption, breakfast cereal, physical activity, treatment arm, atrial fibrilation, diabetes mellitus, hypercholesterolemia, parental history of premature myocardial infarction
Djoussé [23]	PHS, 1981 (US)	20 years	21,327 men (40–85 years)	1084 heart failure events	FFQ	Age, BMI, smoking, alcohol consumption, physical activity, history of diabetes mellitus, atrial fibrillation, hypertension, valvular heart disease, and treatment for cholesterol
Nettleton [24]	ARIC, 1987–1989 (US)	13.3 years	14,153 men and women (45–64 years)	1140 heart failure events	Repeated FFQ	Energy intake, age, sex, race/center, education level, physical activity level, smoking, drinking status, and prevalent disease status: cardiovascular disease, diabetes, and hypertension
Bernstein [25]	NHS, 1980 (US)	26 years	84,136 women (30–55 years)	2210 CHD and 952 CHD death events	Repeated FFQ	Age, time period, total energy, cereal fiber, alcohol, trans fat, BMI, cigarette smoking, menopausal status, parental history of early myocardial infarction, multivitamin use, vitamin E supplement use, aspirin use at least once per week, physical exercise
Scrafford [26]	NHANES III, 1988–1994 (US)	12.2 years	6833 men and 8113 women (≥ 17 years)	168 CHD and 74 stroke events in women and 198 CHD and 63 stroke events in men	FFQ	Age, energy, marital status, educational status, race/ethnicity, smoking status, BMI, WHR, diabetes, hypertension and dietary variables
Bernstein [28]	HPFS, 1986 and NHS, 1980 (US)	26 years in women and 22 years in men	84,010 women (30–55 years) and 43,150 men (40–75 years)	2633 stroke events in women and 1397 stroke events in men	Repeated FFQ	Age, time period BMI, cigarette smoking, physical exercise, parental history of early myocardial infarction, menopausal status in women, multivitamin use, vitamin E supplement use, aspirin use, total energy, cereal fiber, alcohol, transfat, fruit and vegetables, and other protein sources
Houston [49]	Health ABC, 1997–1998 (US)	9 years	1941 men and women (70–79 years)	203 CVD events	FFQ	Age, gender, race, education, field center, smoking, alcohol use, physical activity, BMI, total energy intake, protein intake, fiber intake, multivitamin use, supplemental vitamin E use, statin use, aspirin use, oral estrogen use (women only), prevalent hypertension, and saturated fat
Zazpe [27]	SUN, 1999 (Spain)	6.1 years	14,185 men and women (20–90 years)	91 CVD events	FFQ	Age, sex, total energy intake, adherence to the Mediterranean food pattern, alcohol intake, baseline BMI, smoking status, physical activity during leisure time, family history of CVD, self-reported diabetes, self-reported hypertension, self-reported hypercholesterolemia
Dilis [29]	EPIC-Greece, 1994–1999 (Greece)	10 years	23,929 men and women (20–86 years)	636 CHD events	FFQ	Age, BMI, height, physical activity, years of schooling, energy intake, alcohol consumption, smoking status and arterial blood pressure, and nutritional variables
Misirli [30]	EPIC-Greece, 1994–1999 (Greece)	10.6 years	23,601 men and women (20–86 years)	395 stroke events	FFQ	Sex, age, education, smoking status, BMI, level of physical activity as measured in metabolic equivalents, hypertension, diabetes, and total energy intake
Yaemsiri [31]	WHI-OS, 1994–1998 (US)	7.6 years	87,025 women (50–79 years)	1049 ischemic stroke events	Repeated FFQ	Age, race, education, family income, years as a regular smoker, hormone replacement therapy use, total metabolic equivalent task hours per week, alcohol intake, history of coronary heart disease, history of atrial fibrillation, history of diabetes, aspirin use, use of antihypertensive medication, use of cholesterol-lowering medication, BMI, systolic blood pressure, and total energy intake, dietary vitamin E, fruits and vegetable intake, fiber
Goldberg [32]	NMS, NR (US)	11 years	1429 men and women (> 40 years)	719 CVD (266 stroke events, 226 MI, 452 CVD death events)	FFQ	Age, sex, race/ethnicity, BMI, diabetes, hypertension, LDL, HDL, TG, cholesterol-lowering medication, moderate alcohol use, moderate-heavy physical activity, smoking, high-school completion, daily kcal, Mediterranean diet score, history of stroke, myocardial infarction, daily consumption of saturated fat, unsaturated fat, carbohydrates, and protein
Haring [33]	ARIC, 1987–1989 (US)	22 years	12,066 men and women (45–64 years)	1147 CHD events	Repeated FFQ	Age, sex, race, study center, total energy intake, smoking, cigarette years, education, systolic blood pressure, use of antihypertensive medication, high-density lipoprotein cholesterol, total cholesterol, use of lipid-lowering medication, BMI, waist-to-hip ratio, alcohol intake, sports-related physical activity, leisure-related physical activity, carbohydrate intake, fiber intake, fat intake, and magnesium intake
Haring [34]	ARIC, 1987–1989 (US)	22.7 years	11,601 men and women (45–64 years)	699 stroke events	Repeated FFQ	Age, sex, race, study center, total energy intake, smoking, cigarette years, education, systolic blood pressure, use of antihypertensive medication, high-density lipoprotein cholesterol, total cholesterol, use of lipid-lowering medication, BMI, waist-to-hip ratio, alcohol intake, sports-related physical activity, leisure-related physical activity, carbohydrate intake, fiber intake, fat intake, and magnesium intake
Larsson [35]	COSM, 1997 and SMC, 1987–1990 (Norway)	13 years	37,766 men (45–79 years) and 32,805 women (49–83 years)	1628 HF, 3262 MI, 2039 ischemic strokes, 405 hemorrhagic stroke events in men and 1207 HF, 1504 MI, 1561 ischemic stroke, and 294 hemorrhagic stroke events in women	FFQ	Age, education, family history of myocardial infarction, smoking status and pack-years of smoking, aspirin use, walking/bicycling, exercise, BMI, history of hypertension, hypercholesterolemia, and diabetes, intakes of total energy, alcohol, fruit and vegetables, and processed meat
Farvid [38]	GCS, 2004 (Iran)	11 years	42,403 men and women (36–85 years)	1467 CVD, 764 CHD, 507 stroke events	FFQ	Sex, age, ethnicity, education, marital status, residency, smoking, opium use, alcohol, BMI, systolic blood pressure, occupational physical activity, family history of cancer, wealth score, medication, and energy intake
Virtanen [36]	KIHD, 1984–1989 (Finland)	20.8 years	1032 men (42–60 years)	230 CHD events	4-d food records	Age, examination year, and energy intake, smoking, BMI, diabetes, hypertension, leisure-time physical activity, coronary artery disease history in close relatives, education, and intakes of alcohol, fruit, berries, vegetables, fiber, PUFAs, and SFAs
Díez-Espino [37]	PREDIMED, 2003–2009 (Spain)	5.8 years	7216 men and women (55–80 years)	342 CVD events	FFQ	Age, sex, BMI, intervention group, recruitment center, smoking status, physical activity during leisure time, and educational status, diabetes, hypertension, hypercholesterolemia, family history of CVD, Mediterranean food pattern, alcohol intake, and total energy intake
Guo [39]	CAPS, 1979–1983 and NDNS, 2008–2009 (UK)	22.8 years	2512 men (45–59 years)	715 CVD (248 stroke, 477 MI, 201 heart failure) events	7-d food records	Age, BMI, total energy intake, alcohol consumption, smoking status, energy expenditure, social class, family history of myocardial infarction, diabetes mellitus, sugar intake, fruit consumption, red meat consumption and fiber (cereal and vegetable sources)
Jang [40]	KGES, 2001–2002 (Korea)	7.3 years	9248 men and women (40–69 years)	570 CVD events	FFQ	Age, sex, educational level, residential area, monthly household income, alcohol drinking, smoking in pack-years, physical activity level, dietary supplement use, history of hypertension and dyslipidemia, and the intake levels of total energy, total vegetables, total fruits, red meat, fiber, vitamin E, BMI
Qin [41]	CKB, 2004–2008 (China)	8.9 years	461,213 men and women (30–79 years)	83,977 CVD (30,169 IHD, 7078 hemorrhagic stroke, and 27,745 ischemic stroke) and 9985 CVD death events (3374 IHD, 3435 hemorrhagic stroke, and 1 003 ischemic stroke deaths)	Repeated FFQ	Age at recruitment, sex, education level, household income, marital status, alcohol consumption, tobacco smoking, physical activity in MET-hours/day, BMI, waist-to-hip ratio, prevalent hypertension, use of aspirin, family history of CVD, intake of multivitamin supplementation and dietary pattern
Xu [43]	GBCS, 2003–2008 (China)	9.8 years	28,024 men and women (> 50 years)	873 CVD, 388 IHD and 341 stroke death events	FFQ	Sex, age, education, occupation, family income, smoking status, physical activity, alcohol drinking, self-rated health and chronic disease history (diabetes, hypertension and dyslipidemia), dietary variables (daily dietary energy and vegetable, fruit, milk and nut intake were included in this model with additional adjustment for total energy, vegetable, fruit, milk and nut intake; only in 18,707 participants)
Zamora‐Ros [44]	EPIC-Spain, 1992–1996 (Spain)	18 years	40,621 men and women (29–69 years)	761 CVD death and 184 stroke events	FFQ	center, age at recruitment in 5 year categories, sex, smoking intensity, BMI, lifetime alcohol intake, education level, physical activity, energy intake, and adherence to Mediterranean diet
Abdollahi [42]	KIHD, 1984–1989 (Finland)	21.2 years	1950 men (42–60 years)	217 stroke (166 ischemic and 55 hemorrhagic) events	4-d food records	Age, year of examination, energy intake, BMI, pack-years of smoking, leisure-time physical activity, hypertension medication, intakes of alcohol, fruit, berries, and vegetables
Djoussé [47]	MVP, 2011 (US)	3.24 years (mean)	188,267 men and women (64.4 years mean)	10,160 MI events	FFQ	Age, sex race, education, BMI, exercise, smoking, alcohol intake, and dietary approach to stop hypertension score
Key [50]	EPIC, 1992–2000 (Europe)	12.6 years (mean)	409,885 men and women (~ 55 years)	7198 CHD events	FFQ	Age, smoking status and number of cigarettes per day, history of diabetes mellitus, previous hypertension, prior hyperlipidemia, Cambridge physical activity index, employment status, level of education completed, BMI, current alcohol consumption, and observed intakes of energy, fruit and vegetables combined, sugars, fiber from cereals, and stratified by sex and EPIC center
van den Brandt [53]	NLCS, 1986, (The Netherlands)	~ 9 years	3202 subcohort men and women (55–69 years)	2985 CVD death events	FFQ	Age at baseline, sex, cigarette smoking status, number of cigarettes smoked per day, and years of smoking, history of physician-diagnosed hypertension and diabetes, body height, BMI, non-occupational physical activity, highest level of education, intake of alcohol, vegetables and fruit, energy, use of nutritional supplements, and, in women, postmenopausal HRT
Zhong [45]	Pooled cohorts (from US)^a^	17.7 years	29,615 men and women (mean age 51.6 years at baseline)	5400 CVD events	Harmonized assessment	Age, sex, race/ ethnicity, education, total energy, smoking status, smoking pack- years, cohort-specific physical activity z score, alcohol intake, use of hormone therapy, BMI, diabetes status, systolic blood pressure, use of antihypertensive medications, high-density lipoprotein (HDL) cholesterol, non-HDL cholesterol, and use of lipid-lowering medications, dietary cholesterol consumption
Dehghan [46]	PURE, 2003, (multinational); ONTARGET/TRANSCEND, 2001–2004 (multinational)	9.5 years PURE; 56 months ONTARGET/TRANSCEND	PURE: 114,615 men and women (~ 50 years); ONTARGET/ TRANSCEND 31,410 men and women (≥ 55 years)	PURE: 3410 CVD death events, 8477 CVD events, 3664 MI, 3916 stroke, 939 heart failure; ONTARGET/ TRANSCEND: 2264 CVD death events, 5181 CVD events, 1554 MI, 1394 stroke, 1337 heart failure	FFQ	PURE: age, sex, smoking, location, education, physical activity, history of diabetes, daily intakes of fruits, vegetables, dairy, red meat, poultry, and fish; percentage energy from carbohydrate; total daily energy; and center as a random effect ONTARGET/TRANSCEND: age, sex, smoking, location, BMI, education, physical activity, history of diabetes, history of myocardial infarction; history of stroke; medication; trial allocation; daily intakes of fruit, vegetables, red meat, poultry, fish, and dairy; and regions as a random effect
Drouin-Chartier [48]	HPFS, 1986, NHS, 1980, NHS II, 1991 (US)	32 years (up to)	HPFS: 42,055 men (40–75 years); NHS: 83,349 women (30–55 years); NHS II: 90,214 (25–44 years)	HPFS: 6170 CVD, 4461 CHD, 1740 stroke events; NHS: 7411 CVD, 3896 CHD, 3587 stroke events; NHS II: 1225 CVD, 653 CHD, 576 stroke events	FFQ	Age, race, family history of myocardial infarction, baseline hypercholesterolemia, baseline hypertension, smoking status, BMI, physical activity, oral contraceptive use (in NHS II only), postmenopausal hormone use (in NHS and NHS II only), alcohol intake, and multivitamin use, hypercholesterolemia and hypertension, cumulative average of daily intake of total calories, full fat milk, bacon, unprocessed red meat, other processed meats, refined grains, fruits, vegetables, potatoes, coffee, fruit juices, and sugar-sweetened beverages
Tong [51]	EPIC, 1992–2000 (Europe)	12.7 years (mean)	418,329 men and women (~ 55 years)	7378 stroke events (4281 ischemic and 1430 hemorrhagic)	FFQ	Age, smoking status and number of cigarettes per day, history of diabetes, prior hypertension, prior hyperlipidemia, Cambridge physical activity index, employment status, level of education completed, current alcohol consumption, BMI, and observed intake of energy, and stratified by sex and EPIC center
Xia [54]	China-MUCA, 1998; InterAISA, 200–2001 (China); CIMIC, 2007–2008 (China)	15 years China-MUCA (median); 13 years InterAISA (median); 6 years CIMIC (median)	China-MUCA: 10,410 men and women (35–59 years); InterAISA: 12,660 men and women (35–74 years); CIMIC: 79,066 men and women (≥ 18 years)	Overall 4848 CVD, 1273 CHD, 2919 stroke (1832 ischemic, 862 hemorrhagic) events	FFQ	Age, gender, urban or rural resident, per-capita household income, education attainment, tobacco smoking, alcohol consumption, family history of CVD, physical activity, BMI and dietary factors (red meat intake, fresh fruit and vegetable intake)

*ARIC* Atherosclerosis Risk in Communities, *CAPS* Caerphilly Prospective Cohort Study, *CKB* China Kadoorie Biobank, *COSM* Cohort of Swedish Men, *EPIC* European Prospective into Cancer and Nutrition, *GBCS* Guangzhou Biobank Cohort Study, *GCS* Golestan Cohort Study, *Health ABC* Health, Aging and Body Composition, *HPFS* Health Professionals Follow-up Study, *JPHC* Japan Public Health Center-based prospective study, *KGES* Korean Genome and Epidemiology Study, *KIHD* Kuopio Ischaemic Heart Disease Risk Factor Study, *LSS* Life Span Study, *MVP* Million Veteran Program, *NDNS *National Diet and Nutritional Survey, *NHANES* National Health and Nutrition Examination Survey, *NHS* Nurses’ Health Study, *NIPPON DATA80* Non-communicable Disease and Its Trends in the Aged, 1980, *NMS* Northern Manhattan Study, *NR* not reported, *ONTARGET* Ongoing Telmisartan Alone and in Combination with Ramipril Global End Point Trial, *PHS* Physicians’ Health Study, *PREDIMED* PREvencion con DIeta MEDiterranea, *PURE* Prospective Urban Rural Epidemiology, *SMC* Swedish Mammography Cohort, *SUN* Seguimiento Universidad de Navarra, *TRANSCEND* Telmisartan Randomized Assessment Study in ACEI Intolerant Subjects with Cardiovascular Disease, *WHI-OS* Women’s Health Initiative Observational Study

^a^Cohorts included were Atherosclerosis Risk in Communities (ARIC) Study, Coronary Artery Risk Development in Young Adults (CARDIA) Study, Framingham Heart Study (FHS), Framingham Offspring Study (FOS), Jackson Heart Study (JHS), and the Multi-Ethnic Study of Atherosclerosis (MESA)

### Egg consumption and cardiovascular outcomes

**Fig. 2:**
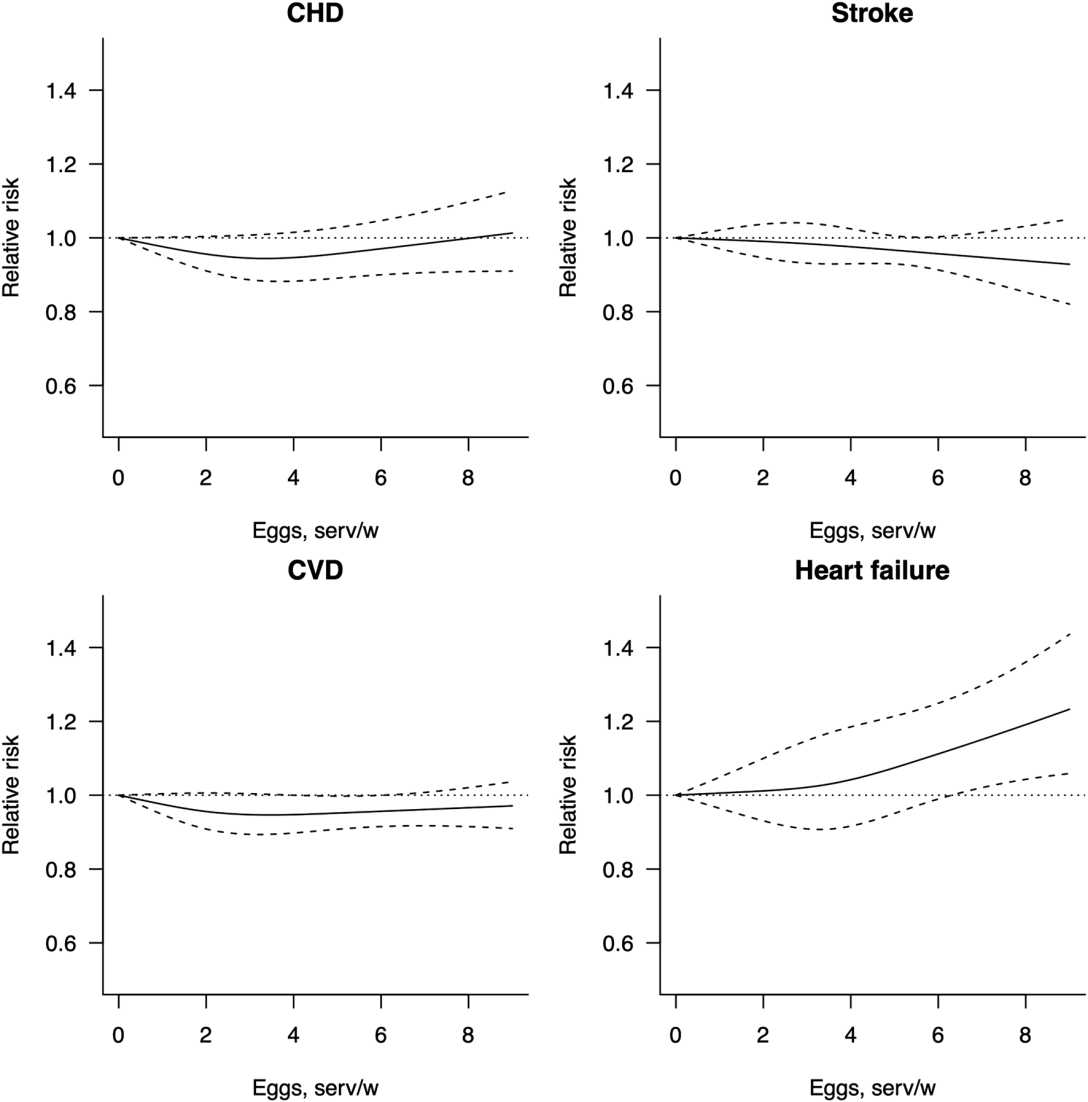
Graphical representation of dose–response association between egg intake and CVD, CHD, stroke and heart failure risk in prospective cohort studies

The dose–response analyses for egg consumption and cardiovascular outcomes are showed in Fig. 2. The summary analysis including 17 datasets from 14 studies conducted on CVD (incidence and/or mortality) showed that intake of up to six eggs per week is inversely associated with CVD events, when comparing to no consumption [SRR = 0.98 (95% CI: 0.95; 1.00), SRR = 0.96 (95% CI: 0.91; 1.00), SRR = 0.95 (95% CI: 0.89; 1.00), SRR = 0.95 (95% CI: 0.90; 1.00), SRR = 0.95 (95% CI: 0.91; 1.00), SRR = 0.96 (95% CI: 0.92; 1.00) for 1, 2, 3, 4, 5, and six eggs per week, respectively; (*I*^2^ = 71.94%, *P*_heter_ < 0.001)] with no evidence of publication bias (*P*_Egger_ = 0.772). The analysis restricted to CVD mortality showed wide confidence intervals while a decreased risk of CVD incidence was observed for consumption of up to 1 egg per day (Table 2).

**Table 2:** Dose–response meta-analysis of the association between egg consumption and cardiovascular outcomes in prospective cohort studies

Outcome	Datasets (studies)	Eggs/week, RR (95% CI)										*I*^*2*^	*P*_het_	*P*_nonlinearity_	*P*_Egger_
		0	1	2	3	4	5	6	7	8	9				
CVD incidence/mortality	17 (14)	1 (ref.)	0.98 (0.95; 1)	0.96 (0.91; 1.00)	0.95 (0.89; 1.00)	0.95 (0.9; 1.00)	0.95 (0.91; 1.00)	0.96 (0.92; 1.00)	0.96 (0.92; 1.01)	0.97 (0.91; 1.02)	0.97 (0.91; 1.04)	71	< 0.001	0.167	0.772
CVD incidence	12 (9)	1 (ref.)	0.97 (0.94; 1)	0.94 (0.89; 1)	0.93 (0.87; 0.99)	0.93 (0.87; 0.99)	0.93 (0.88; 0.98)	0.94 (0.89; 0.99)	0.94 (0.89; 0.99)	0.95 (0.89; 1.01)	0.95 (0.89; 1.03)	75	< 0.001	0.13	0.955
CVD mortality	9 (8)	1 (ref.)	0.98 (0.94; 1.03)	0.97 (0.9; 1.05)	0.96 (0.89; 1.05)	0.96 (0.89; 1.04)	0.96 (0.89; 1.03)	0.95 (0.88; 1.03)	0.95 (0.88; 1.03)	0.95 (0.87; 1.04)	0.95 (0.86; 1.04)	57	0.002	0.667	0.415
CHD incidence/mortality	24 (16)	1 (ref.)	0.98 (0.95; 1)	0.96 (0.91; 1)	0.95 (0.89; 1.01)	0.95 (0.88; 1.02)	0.96 (0.89; 1.03)	0.97 (0.9; 1.05)	0.98 (0.91; 1.07)	1 (0.91; 1.1)	1.01 (0.91; 1.13)	82	< 0.001	0.042	0.173
CHD incidence	17 (12)	1 (ref.)	0.97 (0.94; 1)	0.94 (0.89; 0.99)	0.92 (0.86; 1)	0.93 (0.85; 1.01)	0.94 (0.86; 1.02)	0.96 (0.88; 1.05)	0.98 (0.89; 1.08)	1 (0.9; 1.11)	1.02 (0.9; 1.15)	85	< 0.001	0.011	0.222
CHD mortality	8 (6)	1 (ref.)	1.01 (0.97; 1.06)	1.02 (0.94; 1.11)	1.02 (0.91; 1.14)	1.01 (0.88; 1.15)	0.99 (0.85; 1.15)	0.97 (0.81; 1.16)	0.95 (0.76; 1.18)	0.93 (0.72; 1.2)	0.91 (0.68; 1.23)	1	0.435	0.304	0.542
Stroke incidence/mortality	22 (16)	1 (ref.)	1 (0.97; 1.02)	0.99 (0.95; 1.04)	0.98 (0.93; 1.04)	0.98 (0.93; 1.02)	0.97 (0.93; 1.01)	0.96 (0.91; 1.01)	0.95 (0.88; 1.01)	0.94 (0.85; 1.03)	0.93 (0.82; 1.05)	46	< 0.001	0.842	0.936
Stroke incidence	14 (10)	1 (ref.)	0.99 (0.96; 1.02)	0.97 (0.92; 1.03)	0.96 (0.9; 1.03)	0.96 (0.91; 1.02)	0.97 (0.92; 1.01)	0.97 (0.93; 1.02)	0.98 (0.91; 1.04)	0.98 (0.9; 1.07)	0.99 (0.88; 1.11)	42	0.011	0.503	0.127
Stroke mortality	8 (6)	1 (ref.)	1.03 (0.98; 1.08)	1.05 (0.96; 1.14)	1.04 (0.95; 1.14)	1 (0.93; 1.07)	0.95 (0.86; 1.04)	0.9 (0.77; 1.05)	0.86 (0.69; 1.06)	0.81 (0.62; 1.08)	0.77 (0.55; 1.1)	38	0.063	0.174	0.727
Stroke, ischemic incidence	11 (9)	1 (ref.)	0.99 (0.96; 1.02)	0.98 (0.93; 1.04)	0.97 (0.91; 1.05)	0.97 (0.91; 1.04)	0.97 (0.92; 1.04)	0.98 (0.93; 1.03)	0.98 (0.93; 1.03)	0.98 (0.93; 1.04)	0.99 (0.93; 1.05)	69	< 0.001	0.584	0.855
Stroke, hemorrhagic incidence	10 (8)	1 (ref.)	0.97 (0.93; 1.02)	0.95 (0.88; 1.03)	0.94 (0.84; 1.04)	0.94 (0.84; 1.05)	0.95 (0.84; 1.07)	0.96 (0.83; 1.12)	0.98 (0.8; 1.19)	0.99 (0.78; 1.27)	1.01 (0.75; 1.36)	88	< 0.001	0.347	0.919
Heart failure	6 (4)	1 (ref.)	1.01 (0.96; 1.05)	1.01 (0.93; 1.1)	1.02 (0.91; 1.15)	1.04 (0.92; 1.19)	1.07 (0.95; 1.21)	1.11 (0.99; 1.25)	1.15 (1.02; 1.30)	1.19 (1.04; 1.36)	1.23 (1.06; 1.44)	37	0.103	0.409	0.630

The summary analysis for CHD incidence/mortality including 24 datasets from 16 studies showed a decreased risk up to two eggs per week [SRR = 0.96 (95% CI: 0.91; 1.00), *I*^2^ = 82.25%, *P*_heter_ < 0.001] compared to no consumption, while higher intake was associated with no further reduced risk; no publication bias was detected (*P*_Egger_ = 0.173). Distinction between studies on CHD incidence or mortality showed that the associated reduced risk was referring to the former, yet with evidence of heterogeneity (Table 2).

The summary analysis for stroke including 22 datasets from 16 studies showed no related risks associated with any dose of egg consumption compared to no consumption, with lower SRRs for stroke mortality, though with large CIs (Table 2). Also, the analyses conducted on sub-types of stroke, despite investigated in a lower number of studies (eight studies on hemorrhagic and nine studies on ischemic stroke), showed null associations with egg consumption, yet with evidence of heterogeneity (Table 2).

The summary analysis for heart failure risk including six datasets from four studies showed that intake of one egg per day was associated with increased risk raising for higher intakes compared to no consumption [SRR = 1.15 (95% CI:1.02; 1.30), SRR = 1.19 (95% CI: 1.04; 1.36), SRR = 1.23 (95% CI: 1.06; 1.44) for 7, 8, and nine eggs per week, respectively], with no evidence of heterogeneity (*I*^2^ = 37%) and no publication bias (*P*_Egger_ = 0.630).

In the sensitivity analyses by excluding one study at the time, results were substantially unchanged (data not shown). Also in the sensitivity analyses excluding studies with no complete data on number of individuals and cases risk estimates associated to egg consumption were unchanged for CVD and heart failure, while no associations with CHD and stroke were detected (ESM Table 3); moreover, both ischemic and hemorrhagic stroke risk was reduced with up to one egg per day compared to no consumption (ESM Table 3).

**Table 3:** Subgroup analyses for the association between 4 eggs/week and 1 egg/day consumption and CVD, CHD, stroke, and heart failure

	CVD				CHD				Stroke				Heart failure			
	Datasets (studies)	RR (95% CI)	*I*^2^ (%)	*p*_het_	Datasets (studies)	RR (95% CI)	*I*^2^ (%)	*p*_het_	Datasets (studies)	RR (95% CI)	*I*^2^ (%)	*p*_het_	Datasets (studies)	RR (95% CI)	*I*^2^ (%)	*p*_het_
4 eggs/week																
Sex																
Men	2 (2)	1.04 (0.92; 1.17)	0	0.398	7 (7)	1.03 (0.96; 1.1)	2	0.426	7 (7)	0.99 (0.91; 1.08)	57	0.006	3 (3)	1.05 (0.94; 1.18)	0	0.449
Women	2 (1)	0.89 (0.82; 0.96)	0	0.772	5 (4)	0.89 (0.82; 0.98)	0	0.884	5 (4)	1.05 (0.86; 1.3)	33	0.153	1 (1)	0.84 (0.71; 1)	0	NA
Diabetic individuals	6 (6)	1.22 (0.88; 1.71)	63	0.002	4 (4)	1.19 (0.82; 1.74)	80	< 0.001	5 (5)	1.22 (0.84; 1.77)	57	0.016	1 (1)	1.43 (0.92; 2.22)	0	NA
1 egg/day																
Sex																
Men	2 (2)	1.09 (0.89; 1.34)	0	0.398	7 (7)	0.99 (0.89; 1.09)	2.07	0.426	7 (7)	0.94 (0.75; 1.18)	57	0.006	3 (3)	1.21 (1.01; 1.45)	0	0.449
Women	2 (1)	0.9 (0.81; 1.00)	0	0.772	5 (4)	0.92 (0.81; 1.04)	0.00	0.884	5 (4)	1.04 (0.83; 1.32)	33	0.153	1 (1)	0.94 (0.75; 1.18)	0	NA
Diabetic individuals	6 (6)	1.22 (1.08; 1.39)	63	0.002	4 (4)	1.25 (0.89; 1.75)	80	< 0.001	5 (5)	1.33 (0.87; 2.05)	57	0.016	1 (1)	1.35 (0.85; 2.13)	0	NA

### Subgroup analyses

The subgroup analysis including results on cohort restricted to diabetic individuals showed that the direction of the risk associated with egg consumption was substantially inverted for all outcomes (Table 3); among all of them, the risk of CVD incidence/mortality peaked up to one egg per day [SRR = 1.22 (95% CI: 1.08; 1.39), *I*^2^ = 63%)] compared to no consumption.

The subgroup analysis by sex revealed a different relation with risk of CVD, CHD, and heart failure in women than in men. Among women a decreased risk of all outcomes for consumption of 4 eggs per week [SRR = 0.89 (95% CI: 0.82; 0.96), SRR = 0.89 (95% CI: 0.82; 0.98), and 0.84 (95% CI: 0.71; 1.00), respectively] with no evidence of heterogeneity between studies was observed (Table 3). When considering consumption of one egg per day, only CVD risk was decreased in women [SRR = 0.90 (95% CI: 0.81; 1.00)], with no evidence of heterogeneity (*I*^2^ = 0%) (Table 3). No significant associations were observed among men (Table 3).

### Stratified analyses

Several stratified analyses have been performed to test the stability of results taking into consideration the geographical localization of the cohorts as well as the level of adjustment models and the quality of the studies included. The analyses have been considered for a moderate consumption (four eggs per week) and a habitual consumption (one egg per day). Concerning the intake of 4 eggs/week, the decreased risk of CVD was confirmed when restricting the analyses to the majority of better quality studies, such as those adjusting for BMI, other dietary factors, longer follow-up, larger sample size, including heart failure in the definition of CVD, and scoring moderate risk of bias (Table 4); other strata associated with a decreased risk of CVD where studies conducted in US cohorts. Similar associations were retrieved for risk of CHD, with a direction toward reduction when restricting the analysis to studies adjusting for other dietary factors, longer follow-up and low risk of bias (Table 4). No association between moderate egg consumption and risk of stroke nor heart failure was found (Table 4).

**Table 4:** Stratified analyses for the association between 4 eggs/week and 1 egg/day consumption and CVD, CHD, stroke, and heart failure

	CVD				CHD				Stroke				Heart failure			
	Datasets (studies)	RR (95% CI)	*I*^2^ (%)	*p*_het_	Datasets (studies)	RR (95% CI)	*I*^2^ (%)	*p*_het_	Datasets (studies)	RR (95% CI)	*I*^2^ (%)	*p*_het_	Datasets (studies)	RR (95% CI)	*I*^2^ (%)	*p*_het_
4 eggs/week																
Location																
USA	5 (3)	0.94 (0.89; 0.99)	20	0.264	10 (6)	0.98 (0.89; 1.09)	70	< 0.001	9 (6)	0.98 (0.91; 1.04)	56	0.002	1 (1)	1.05 (0.88; 1.26)	0	NA
Europe	5 (5)	1.07 (0.94; 1.22)	0	0.883	5 (4)	0.98 (0.92; 1.03)	0	0.756	5 (4)	1.01 (0.9; 1.14)	27	0.204	3 (2)	0.99 (0.81; 1.21)	29	0.225
Asia	5 (5)	0.93 (0.84; 1.04)	90	< 0.001	7 (5)	0.92 (0.77; 1.09)	90	< 0.001	6 (5)	0.93 (0.81; 1.07)	63	0.002	–	–	–	–
Adjusted for BMI																
No	2 (2)	0.97 (0.76; 1.24)	78	0.010	2 (2)	0.95 (0.56; 1.6)	89	< 0.001	2 (2)	0.99 (0.89; 1.09)	13	0.313	1 (1)	1.05 (0.82; 1.35)	0	NA
Yes	15 (13)	0.94 (0.89; 0.99)	72	< 0.001	22 (15)	0.95 (0.89; 1.02)	82	< 0.001	20 (15)	0.98 (0.92; 1.03)	48	0.001	5 (4)	1.04 (0.9; 1.22)	39	0.104
Adjusted for diabetic status																
No	8 (6)	0.91 (0.86; 0.96)	80	< 0.001	7 (5)	0.93 (0.81; 1.06)	93	< 0.001	7 (5)	0.91 (0.82; 1.01)	18	0.259	–	–	–	–
Yes	9 (8)	0.98 (0.9; 1.07)	48	0.014	17 (12)	0.96 (0.89; 1.04)	50	< 0.001	15 (11)	1.01 (0.96; 1.06)	49	0.002	6 (4)	1.04 (0.92; 1.19)	37	0.103
Adjusted for other dietary factors																
No	3 (3)	0.98 (0.86; 1.11)	63	0.028	6 (5)	1.06 (0.95; 1.19)	42	0.065	7 (6)	0.99 (0.91; 1.08)	30	0.142	1 (1)	1.05 (0.88; 1.26)	0	NA
Yes	14 (11)	0.94 (0.88; 1)	72	< 0.001	18 (13)	0.92 (0.85; 0.99)	84	< 0.001	15 (10)	0.96 (0.91; 1.02)	51	0.001	5 (3)	1.05 (0.88; 1.24)	35	0.137
Follow-up, years																
< 10	7 (6)	0.99 (0.89; 1.09)	62	0.002	5 (4)	0.94 (0.77; 1.16)	94	< 0.001	3 (2)	1.03 (0.91; 1.17)	29	0.228	2 (1)	1.17 (0.94; 1.46)	2	0.359
> = 10	10 (8)	0.93 (0.87; 1)	43	0.024	19 (13)	0.94 (0.89; 0.99)	62	< 0.001	19 (14)	0.96 (0.92; 1.02)	47	0.001	4 (3)	0.99 (0.86; 1.15)	35	0.159
Sample size																
< 10,000	4 (4)	1.06 (0.9; 1.25)	0	0.418	7 (5)	1.08 (0.86; 1.34)	0	0.790	7 (5)	1.16 (0.91; 1.47)	69	< 0.001	1 (1)	1.38 (0.81; 2.34)	0	NA
> = 10,000	13 (10)	0.94 (0.89; 1)	74	< 0.001	17 (12)	0.94 (0.87; 1.01)	87	< 0.001	15 (11)	0.98 (0.92; 1.03)	24	0.116	5 (3)	1.02 (0.9; 1.16)	40	0.094
Study quality																
Moderate risk of bias	12 (10)	0.95 (0.91; 0.99)	27	0.115	16 (11)	0.95 (0.88; 1.02)	82	< 0.001	13 (9)	0.98 (0.93; 1.03)	38	0.03	4 (3)	0.99 (0.86; 1.15)	35	0.159
Serious risk of bias	5 (4)	0.95 (0.82; 1.09)	79	< 0.001	8 (6)	0.92 (0.75; 1.12)	66	< 0.001	9 (7)	0.97 (0.87; 1.07)	59	0.001	2 (1)	1.17 (0.94; 1.46)	2	0.359
Inclusion of HF in CVD outcome																
No	7 (7)	1.01 (0.94; 1.09)	5	0.399	–	–	–	–	–	–	–	–	–	–	–	–
Yes	7 (7)	0.89 (0.84; 0.94)	84	< 0.001	–	–	–	–	–	–	–	–	–	–	–	–
1 egg/day																
Location																
USA	5 (3)	0.97 (0.9; 1.05)	20	0.264	10 (6)	1.05 (0.98; 1.13)	70	< 0.001	9 (6)	0.96 (0.79; 1.16)	56	0.002	1 (1)	1.32 (1.11; 1.58)	0	NA
Europe	5 (5)	1.17 (0.95; 1.43)	0	0.883	5 (4)	0.97 (0.86; 1.09)	0	0.756	5 (4)	1.04 (0.82; 1.33)	27	0.204	3 (2)	1.08 (0.88; 1.32)	29	0.225
Asia	5 (5)	0.92 (0.87; 0.98)	90	< 0.001	7 (5)	0.95 (0.74; 1.21)	90	< 0.001	6 (5)	0.86 (0.79; 0.93)	63	0.002	–	–	–	–
Adjusted for BMI																
No	2 (2)	0.97 (0.82; 1.14)	78	0.010	2 (2)	0.98 (0.66; 1.46)	89	< 0.001	2 (2)	0.9 (0.73; 1.11)	13	0.313	1 (1)	1.02 (0.81; 1.29)	0	NA
Yes	15 (13)	0.96 (0.91; 1.01)	72	< 0.001	22 (15)	0.98 (0.90; 1.07)	82	< 0.001	20 (15)	0.96 (0.89; 1.03)	48	0.001	5 (4)	1.19 (1.03; 1.38)	39	0.104
Adjusted for diabetic status																
No	8 (6)	0.9 (0.88; 0.92)	80	< 0.001	7 (5)	0.98 (0.82; 1.17)	93	< 0.001	7 (5)	0.97 (0.85; 1.1)	18	0.259	–	–	–	–
Yes	9 (8)	1 (0.93; 1.07)	48	0.014	17 (12)	0.97 (0.92; 1.02)	50	< 0.001	15 (11)	0.98 (0.86; 1.1)	49	0.002	6 (4)	1.15 (1.02; 1.3)	37	0.103
Adjusted for other dietary factors																
No	3 (3)	1.01 (0.93; 1.09)	63	0.028	6 (5)	1.04 (0.92; 1.18)	42	0.065	7 (6)	0.93 (0.85; 1.03)	30	0.142	1 (1)	1.32 (1.11; 1.58)	0	NA
Yes	14 (11)	0.95 (0.9; 1)	72	< 0.001	18 (13)	0.97 (0.88; 1.07)	84	< 0.001	15 (10)	0.95 (0.87; 1.04)	51	0.001	5 (3)	1.11 (0.96; 1.28)	35	0.137
Follow-up, years																
< 10	7 (6)	0.96 (0.9; 1.02)	62	0.002	5 (4)	0.98 (0.79; 1.21)	94	< 0.001	3 (2)	0.97 (0.86; 1.08)	29	0.228	2 (1)	1.16 (0.86; 1.57)	2	0.359
> = 10	10 (8)	0.97 (0.92; 1.03)	43	0.024	19 (13)	0.97 (0.92; 1.03)	62	< 0.001	19 (14)	0.95 (0.88; 1.03)	47	0.001	4 (3)	1.14 (0.98; 1.34)	35	0.159
Sample size																
< 10,000	4 (4)	1.16 (0.82; 1.64)	0	0.418	7 (5)	1.04 (0.79; 1.36)	0	0.790	7 (5)	1.21 (0.63; 2.32)	69	< 0.001	1 (1)	1.14 (0.61; 2.12)	0	NA
> = 10,000	13 (10)	0.96 (0.91; 1.01)	74	< 0.001	17 (12)	0.98 (0.9; 1.07)	87	< 0.001	15 (11)	0.96 (0.91; 1)	24	0.116	5 (3)	1.15 (1; 1.31)	40	0.094
Study quality																
Moderate risk of bias	12 (10)	0.97 (0.91; 1.05)	27	0.115	16 (11)	0.96 (0.88; 1.05)	82	< 0.001	13 (9)	0.99 (0.89; 1.1)	38	0.03	4 (3)	1.14 (0.98; 1.34)	35	0.159
Serious risk of bias	5 (4)	0.97 (0.88; 1.06)	79	< 0.001	8 (6)	1.04 (0.84; 1.28)	66	< 0.001	9 (7)	0.9 (0.81; 0.99)	59	0.001	2 (1)	1.16 (0.86; 1.57)	2	0.359
Inclusion of HF in CVD outcome																
No	7 (7)	1.03 (0.97; 1.1)	5	0.399	–	–	–	–	–	–	–	–	–	–	–	–
Yes	7 (7)	0.9 (0.88; 0.92)	84	< 0.001	–	–	–	–	–	–	–	–	–	–	–	–

Concerning the intake of 1 egg/day, the analysis resulted in a decreased risk of CVD when involving studies conducted in Asia and adjusting for other dietary factors (Table 4); curiously, a decreased risk was also observed in studies not adjusting for diabetic status, which on the contrary was reported to potentially act as effect modifier toward the opposite direction. No associations were retrieved for CHD risk, while also the risk of stroke was reduced only when considering Asian cohorts and studies including more than 10,000 individuals (Table 4). Also risk of heart failure differed between strata, resulting higher in the analysis restricted to US cohorts with large sample size, adjusted for BMI but not for other dietary factors; no study with low risk of bias was available (Table 4).

### Evaluation of the evidence

Table 5 provides an overview of the GRADE assessment for the association between consumption of eggs and each cardiovascular outcome. The level of evidence was rated generally low for all outcomes but stroke, for which was moderate.

**Table 5:** Certainty of evidence by GRADE criteria

	CVD	CHD	Stroke	Heart failure
No. of studies	17 (14)	24 (16)	22 (16)	6 (4)
Downgrade quality of evidence				
Risk of bias	No	No	No	No
Inconsistency	Yes	Yes	No	No
Indirectness	No	No	No	Yes
Imprecision	No	No	No	No
Publication Bias	No	No	No	No
Upgrade quality of evidence				
Large effect	No	No	No	No
Plausible confounding	No^a^	No^a^	No	No
Dose–response	Yes^b^	No	Yes^b^	Yes^b^
Overall quality of evidence	Low	Low	Moderate	Low

^a^Despite better quality studies provided less heterogeneity across results

^b^The analyses showed no evidence for non-linearity of associations

## Discussion

The present meta-analysis provided an updated overview on the association between egg consumption and CVD risk and mortality: compared to previous meta-analyses, we included the highest number of cohorts reviewed to date, several dose–response analyses for the investigated outcomes, a detailed investigation for potential confounding factors by studying subgroups and stratifying the analyses, and we attempted an evaluation of the overall evidence. Previous meta-analyses reported rather mixed results, with no association with stroke risk [55], decreased risk of stroke and no association with CHD [56, 57], decreased risk of CHD [58], no association with CVD risk [59], increased risk of heart failure [60, 61],compared to these studies, our analysis is more complete and provides a general more in depth analysis of level of evidence. We generally found no strong association with either increased or decreased risk of cardiovascular outcomes following the habitual consumption of eggs (i.e., one egg per day compared to no intake), with exception of risk of heart failure, which resulted higher especially in men from US cohorts. In contrast, there are more consistent results regarding the association between moderate egg consumption (i.e., four eggs per week compared to no intake) and lower risk of CVD, especially in spite of the stratified analyses involving higher quality studies, for which there was lower heterogeneity across results. Also when considering the findings from the stratified analyses, heterogeneity of the results between studies remained significant and rather unexplained. We can hypothesize that egg consumption between men and women or across different geographical areas may be associated with unmeasured lifestyle choices or in the context of different quality of the overall diet to motivate the differences observed in these strata, another hypothesis is that these strata may also reflect genetic unmeasured factors motivating the inter-individual variations. After the GRADE assessment, we could not conclude that exist strong evidence of association between egg consumption and CVD outcomes, but higher quality studies showed a decreased risk of CVD for moderate intake of eggs (four per week), while higher risk of heart failure was found for higher intake of egg (one per day). Interpretation of these findings is not easy: consuming up to four eggs per week may decrease the risk of CVD but increasing the intake to one egg per day or more may not be beneficial anymore. Nonetheless, we cannot exclude that the pattern of the diet and way of cooking might differ depending on the frequency of consumption (i.e., individuals with moderate egg consumption may include eggs into specific recipes varying the way of cooking while habitual consumers may have fried eggs for breakfast together with bacon or other unhealthy dietary features). Such hypothesis could explain the different risks associated with egg consumption in Western and Eastern Asian countries. This is a common issue for nearly all food groups when investigating the relation of one single dietary element with health. However, we cannot ignore that an association can be observed and, in that case, needs further attention.

There is a biological rationale to explain how moderate egg consumption might be associated to decreased risk of CVD. Eggs have been historically considered a controversial food for nutritional experts and health agencies due to its content in cholesterol. However, researchers argue that the focus of common dietary guidelines on specific nutrients (i.e., saturated fats) do not take into account that health effects varies depending on the specific food source [62]. Furthermore, the major attention paid to egg consumption has been based on the assumption that higher dietary cholesterol intake would lead to rise in blood cholesterol, despite current evidence suggests otherwise [63, 64]. Recent meta-analyses showed rise of both LDL and HDL following egg consumption in healthy individuals, with minimum rise of LDL:HDL ratio (marker of CVD risk) finally leading to no substantial increased risk profile [63, 64]. Thus, the concomitant rise of HDL cholesterol might counteract the elevation of LDL, while other components of egg might exert potential beneficial effects [65]. Eggs are a highly nutritious food providing quality proteins and supplying micronutrients, antioxidants, antimicrobials, accompanied with great culinary versatility, which may have potential benefits to overall health. Some egg proteins, such as phosvitin, ovotransferrin and ovalbumin can inhibit lipid oxidation by binding to metal or scavenging free radical [66]. In addition to protein, eggs also contain a large number of active lipid components, such as unsaturated fatty acids, phospholipids, choline, and carotenoids. Eggs are considered a valuable source of omega-3 polyunsaturated fatty acids, which have been considered to exert a number of health benefits, including CVD protection [67]. Eggs are a major source of choline, an essential nutrient with critical roles in several biological processes including neuronal development, cell signaling, and lipid transport and metabolism [68]. Part of the choline may undergo conversion to trimethylamine by gut microbiota, which in turn is oxidized in the liver to trimethylamine-*N*-oxide (TMAO), agent associated with increased atherosclerosis in the coronary vasculature [69]. Double blinded clinical trial investigating the effect of 0 to 6 egg yolks ingested for the breakfast demonstrated that consumption of ≥ 2 eggs results in an increased formation of TMAO yet not accompanied by a rise in hsCRP and oxidized LDL levels [70]. Phospholipids contained in eggs, including phosphatidylcholine, phosphatidylethanolamine, lysophosphatidylcholine, sphingomyelin, and some neutral lipids in minor quantities, may have, among others, broad effects on cholesterol metabolism, HDL functions, and inflammation [71]. Egg yolks are also a dietary source of bioavailable xanthophyll carotenoids, such as lutein and zeaxanthin, that have been shown to exert potential benefits against inflammation and oxidation during early development, childhood, and may have lifetime consequences in determining health or onset of major diseases in the adult life [72].

Despite the evidence reported, nearly all analyses showed substantial heterogeneity of results between studies, leading to a weakening of the evidence. We hypothesize that the certain inconsistency of the results may depend on the variability of response to dietary cholesterol between individuals and the overall dietary and lifestyle framework within populations and individuals. Despite the majority of population experience moderate to no difference in blood cholesterol following the intake of dietary cholesterol (consequently described as “normal responders”), about a third of individuals suffer of an abnormal rise in circulating LDL cholesterol (thus described as “hyper responders”) as a result of an increase fractional absorption and/or endogenous cholesterol synthesis in response to dietary cholesterol intake [73]. An abnormal response to dietary cholesterol has been hypothesized to depend on altered cholesterol transport due to decreased levels of apolipoprotein E and increased of apolipoprotein C-III [74, 75]. Some genes responsible for intestinal absorption and biliary secretion of cholesterol and phytosterols, such as expression of ATP-binding cassette (ABC) transporters G5 (ABCG5) and G8 (ABCG8) [76], have been proposed as candidates for better understanding of potential genetic influences on egg metabolism. These genetic variants might provide the rationale, at least in part, for the geographical and sex differences observed in this study: however, further studies are warranted to identify other genetic markers that may explain the observed variability in cholesterol absorption/production among the general population.

The results of this meta-analysis on heart failure seems to provide indication of potential increased risk for habitual consumption (one egg per day) despite this evidence was affected by some limitations, including the role of sex: while sex seems to act as confounding factor for CVD, the observed variation in the direction for risk heart failure (increased in men and decreased in women) may lead to consider sex as an effect modifier. The reasons for such finding is not clear. The role of cholesterol abnormalities and risk or worsening of heart failure is unknown; data on worsening heart failure and lipid moieties are now beginning to emerge but conclusions are far to be made [77]. The fact that heart failure was the only outcome potentially at higher risk following consumption of eggs suggests that alternative mechanisms could be responsible for the observed association. Interpretation of these differences between sexes makes even harder to provide a rationale for this result: as suggested in the individual studies included in the meta-analysis, it may be possible that the observed difference between sexes may depend on the fact that men might be more sensitive to high consumption of eggs (or cholesterol) than women, or it could be mediated by uncontrolled risk factors associated with egg consumption (i.e., bacon) occurring more in men than women. Another hypothesis is that individuals more sensible to dietary cholesterol presenting blood lipids abnormalities may be regular users of statins, which in turn are known to increase the risk of atherosclerosis and heart failure by promoting arteries calcification and inhibiting the biosynthesis of selenium containing proteins, respectively [78]: this might explain the mixed results for CHD risk and increased risk of heart failure.

Another concern regard the different risk estimates observed in diabetic individuals showing an increased risk of CVD associated with consumption of one egg per day, notably in the different direction than for the general population. The increased risk of developing CVD among individuals with type 2 diabetes may be mainly attributed to the impaired cholesterol absorption and synthesis. Studies on type 2 diabetic patients with uncontrolled hyperglycemia showed higher cholesterol synthesis and plasma lipid concentrations [79], including total cholesterol and triglyceride, suggesting unfavorable effects of egg consumption on lipid profiles and, consequently, CVD risk. The mechanism might be explained, at least partially, by a reduced plasma level of campesterol, a marker of cholesterol absorption, and increased plasma levels of lathosterol, a marker of cholesterol synthesis among diabetic people [80]. Moreover, apolipoprotein E polymorphism has been associated with higher risk of diabetes, and thus diabetic individuals tend to have lower serum levels of apolipoptorein E and impaired lipid transport [81].

The findings reported in this study should be considered in light of some limitations. First, some analyses showed substantial heterogeneity: several reasons for such discrepancy of results across studies have been aforementioned, but no firm explanation can be drafted at this moment due to lack of data. Second, we stratified the analyses testing the role of controlling for potential confounding factors known to be related to cardiovascular outcomes in the original studies and revealed the importance of conducting higher quality studies to observe a decreased risk of CVD associated with moderate egg consumption; however, we cannot rule out the possibility that residual or unmeasured confounding may persist. Third, time-related variables, including potential reverse causation (i.e., change in dietary intake due to diagnosed medical condition or disease), period of evaluation (i.e., baseline assessment or repeated over time), or duration of egg intake have been not investigated. Finally, the GRADE system may not be the best suit for assessing evidence in nutritional epidemiology, as by definition it tends to underestimate the strength of the evidence due to the observational nature of the studies. However, it helps to have a clearer idea of which can be the strengths and weaknesses of the studies evaluated (i.e., results from better quality studies are less heterogeneous and tend to show a decreased risk of CVD for moderate egg consumption) and a guide for future investigations.

## Conclusion

Given the inconsistency of current findings on egg consumption and risk of CVD, future studies should improve the characterization of the population investigated, aiming to identify and remove genetic bias, such as the determinants of normal/hyper-response to dietary cholesterol. However, current evidence is not sufficient to address egg consumption as unhealthy nor to generalize potential detrimental effects to the whole population. While waiting for better designed and more complete studies overcoming the aforementioned limitations and lack of information on genetic profile, there may be no need to discourage egg consumption at the population level.

## Electronic supplementary material

Below is the link to the electronic supplementary material.

Supplementary file1 (DOCX 38 kb)
