# Supplementary material for: Egg consumption and cardiovascular risk: a dose–response meta-analysis of prospective cohort studies
Source: Eur J Nutr. 2020 Aug 31;60(4):1833–62. doi: 10.1007/s00394-020-02345-7 (PMC8137614; doi:10.1007/s00394-020-02345-7)
Supplement: Supplementary file 1 — Supplementary file1 (DOCX 38 kb) [file 394_2020_2345_MOESM1_ESM.docx]

**Egg consumption and cardiovascular risk: a dose-response meta-analysis of prospective cohort studies**

**European Journal of Nutrition**

Justyna Godos^1^, Agnieszka Micek^2^, Tomasz Brzostek^3^, Estefania Toledo^4,5,6^, Licia Iacoviello^7,8^, Arne Astrup^9^, Oscar H. Franco^10,11^, Fabio Galvano^12^, Miguel A. Martinez-Gonzalez^4,5,6,13^*, Giuseppe Grosso^12^*

^1^ Oasi Research Institute - IRCCS, Troina, Italy;

^2^ Department of Nursing Management and Epidemiology Nursing, Faculty of Health Sciences, Jagiellonian University Medical College, Krakow, Poland

^3^ Department of Internal Medicine and Community Nursing, Faculty of Health Sciences, Jagiellonian University Medical College, Krakow, Poland;

^4^ University of Navarra, Department of Preventive Medicine and Public Health, School of Medicine, Pamplona, Navarra, Spain;

^5^ CIBER Physiopathology of Obesity and Nutrition (CIBEROBN), Carlos III Institute of Health, Madrid, Spain;

^6^ Navarra Institute for Health Research, IdiSNA, Pamplona, Navarra, Spain;

^7^ Department of Epidemiology and Prevention, IRCCS NEUROMED, Pozzilli (IS), Italy;

^8^ Research Centre in Epidemiology and Preventive Medicine (EPIMED), Department of Medicine and Surgery, University of Insubria, Varese, Italy;

^9^ Department of Nutrition, Exercise, and Sports, Faculty of Science, University of Copenhagen, Nørre Campus, Copenhagen, Denmark;

^10^ Department of Epidemiology, Erasmus MC, University Medical Center Rotterdam, Rotterdam, Netherlands;

^11^ Institute of Social and Preventive Medicine, University of Bern, Bern, Switzerland;

^12^ Department of Biomedical and Biotechnological Sciences, University of Catania, Catania, Italy;

^13^ Department of Nutrition, Harvard T.H. Chan School of Public Health, Boston, MA, USA

* Authors contributed equally

**Corresponding Author**

Giuseppe Grosso MD, PhD, Department of Biomedical and Biotechnological Sciences, University of Catania, Via S. Sofia 97, 95123, Catania, Italy; Fax/Phone: +39 0954781187, Email: [giuseppe.grosso@unict.it](mailto:giuseppe.grosso@unict.it)

**ESM** **Table 1. Moose checklist.**

| **Section/topic** | **#** | **Checklist item** | **Reported on page #** |
| --- | --- | --- | --- |
| **TITLE** | | |  |
| Title | 1 | Identify the report as a systematic review, meta-analysis, or both. | 1 |
| **ABSTRACT** | | |  |
| Structured summary | 2 | Provide a structured summary including, as applicable: background; objectives; data sources; study eligibility criteria, participants, and interventions; study appraisal and synthesis methods; results; limitations; conclusions and implications of key findings; systematic review registration number. | 2,3 |
| **INTRODUCTION** | | |  |
| Rationale | 3 | Describe the rationale for the review in the context of what is already known. | 1 |
| Objectives | 4 | Provide an explicit statement of questions being addressed with reference to participants, interventions, comparisons, outcomes, and study design (PICOS). | 1 |
| **METHODS** | | |  |
| Protocol and registration | 5 | Indicate if a review protocol exists, if and where it can be accessed (e.g., Web address), and, if available, provide registration information including registration number. | NA |
| Eligibility criteria | 6 | Specify study characteristics (e.g., PICOS, length of follow-up) and report characteristics (e.g., years considered, language, publication status) used as criteria for eligibility, giving rationale. | 6 |
| Information sources | 7 | Describe all information sources (e.g., databases with dates of coverage, contact with study authors to identify additional studies) in the search and date last searched. | 6 |
| Search | 8 | Present full electronic search strategy for at least one database, including any limits used, such that it could be repeated. | 6 |
| Study selection | 9 | State the process for selecting studies (i.e., screening, eligibility, included in systematic review, and, if applicable, included in the meta-analysis). | 6 |
| Data collection process | 10 | Describe method of data extraction from reports (e.g., piloted forms, independently, in duplicate) and any processes for obtaining and confirming data from investigators. | 6,7 |
| Data items | 11 | List and define all variables for which data were sought (e.g., PICOS, funding sources) and any assumptions and simplifications made. | 6,7 |
| Risk of bias in individual studies | 12 | Describe methods used for assessing risk of bias of individual studies (including specification of whether this was done at the study or outcome level), and how this information is to be used in any data synthesis. | 7,8 |
| Summary measures | 13 | State the principal summary measures (e.g., risk ratio, difference in means). | 7,8 |
| Synthesis of results | 14 | Describe the methods of handling data and combining results of studies, if done, including measures of consistency (e.g., I^2^) for each meta-analysis. | 7,8 |
| Risk of bias across studies | 15 | Specify any assessment of risk of bias that may affect the cumulative evidence (e.g., publication bias, selective reporting within studies). | 7,8 |
| Additional analyses | 16 | Describe methods of additional analyses (e.g., sensitivity or subgroup analyses, meta-regression), if done, indicating which were pre-specified. | 7,8 |
| **RESULTS** | | |  |
| Study selection | 17 | Give numbers of studies screened, assessed for eligibility, and included in the review, with reasons for exclusions at each stage, ideally with a flow diagram. | 8 |
| Study characteristics | 18 | For each study, present characteristics for which data were extracted (e.g., study size, PICOS, follow-up period) and provide the citations. | Table 1 |
| Risk of bias within studies | 19 | Present data on risk of bias of each study and, if available, any outcome level assessment (see item 12). | Suppl Fig |
| Results of individual studies | 20 | For all outcomes considered (benefits or harms), present, for each study: (a) simple summary data for each intervention group (b) effect estimates and confidence intervals, ideally with a forest plot. | Tables and Figures |
| Synthesis of results | 21 | Present results of each meta-analysis done, including confidence intervals and measures of consistency. | Tables and Figures |
| Risk of bias across studies | 22 | Present results of any assessment of risk of bias across studies (see Item 15). | Suppl Fig |
| Additional analysis | 23 | Give results of additional analyses, if done (e.g., sensitivity or subgroup analyses, meta-regression [see Item 16]). | Suppl Fig |
| **DISCUSSION** | | |  |
| Summary of evidence | 24 | Summarize the main findings including the strength of evidence for each main outcome; consider their relevance to key groups (e.g., healthcare providers, users, and policy makers). | 13-15 |
| Limitations | 25 | Discuss limitations at study and outcome level (e.g., risk of bias), and at review-level (e.g., incomplete retrieval of identified research, reporting bias). | 15 |
| Conclusions | 26 | Provide a general interpretation of the results in the context of other evidence, and implications for future research. | 15,16 |
| **FUNDING** | | |  |
| Funding | 27 | Describe sources of funding for the systematic review and other support (e.g., supply of data); role of funders for the systematic review. | 16 |

**ESM Table 2. Risk of bias assessment of all included studies in the meta-analysis investigating the association between egg consumption and cardiovascular outcomes using the Risk Of Bias In Non-randomized Studies – of Interventions (ROBINS-I) assessment tool.**

|  | Domains of bias | | | | | | | Overall score |
| --- | --- | --- | --- | --- | --- | --- | --- | --- |
| Author, year | Confounding | Selection | Exposure assessment | Follow-up | Missing data | Outcome assessment | Selective reporting |  |
| Hu, 1999 | moderate | moderate | low | low | low | low | moderate | moderate |
| He, 2003 | moderate | moderate | low | low | low | low | moderate | moderate |
| Sauvaget, 2003 | serious | moderate | moderate | moderate | moderate | low | low | serious |
| Nakamura, 2004 | moderate | low | serious | moderate | moderate | low | moderate | serious |
| Nakamura, 2006 | moderate | moderate | low | low | low | moderate | low | moderate |
| Trichopoulou, 2006 | moderate | low | moderate | moderate | no information | low | moderate | moderate |
| Qureshi, 2007 | moderate | low | moderate | moderate | low | low | low | moderate |
| Djoussé, 2008 | moderate | moderate | moderate | moderate | no information | low | moderate | moderate |
| Djoussé, 2008 | moderate | moderate | moderate | moderate | no information | low | moderate | moderate |
| Nettleton, 2008 | serious | moderate | low | low | low | moderate | moderate | serious |
| Bernstein, 2010 | moderate | moderate | low | low | low | low | low | moderate |
| Scrafford, 2010 | moderate | low | moderate | moderate | moderate | low | moderate | moderate |
| Bernstein, 2011 | moderate | moderate | low | low | low | low | low | moderate |
| Houston, 2011 | moderate | moderate | low | moderate | low | low | low | moderate |
| Zazpe, 2011 | moderate | moderate | moderate | moderate | low | low | moderate | moderate |
| Dilis, 2012 | moderate | low | moderate | moderate | low | low | moderate | moderate |
| Misirli, 2012 | moderate | low | moderate | moderate | low | low | low | moderate |
| Yaemsiri, 2012 | moderate | moderate | low | moderate | no information | low | moderate | moderate |
| Goldberg, 2014 | moderate | moderate | serious | moderate | moderate | low | moderate | serious |
| Haring, 2014 | moderate | moderate | low | low | low | moderate | low | moderate |
| Haring, 2015 | moderate | moderate | low | low | low | low | low | moderate |
| Larsson, 2015 | moderate | moderate | moderate | moderate | moderate | low | moderate | moderate |
| Farvid, 2016 | moderate | low | moderate | moderate | low | low | moderate | moderate |
| Virtanen, 2016 | moderate | moderate | serious | moderate | low | low | moderate | serious |
| Díez-Espino, 2017 | moderate | moderate | low | moderate | no information | low | low | moderate |
| Guo, 2018 | moderate | moderate | low | low | moderate | low | moderate | moderate |
| Jang, 2018 | moderate | low | moderate | moderate | no information | moderate | low | moderate |
| Qin, 2018 | moderate | low | low | low | no information | low | low | moderate |
| Xu, 2018 | serious | moderate | moderate | moderate | low | low | low | serious |
| Zamora‐Ros, 2018 | moderate | low | moderate | moderate | no information | low | moderate | moderate |
| Abdollahi, 2019 | moderate | moderate | serious | moderate | low | low | low | serious |
| Djoussé, 2019 | moderate | moderate | moderate | moderate | no information | low | moderate | moderate |
| Key, 2019 | moderate | low | moderate | moderate | low | low | low | moderate |
| van den Brandt, 2019 | moderate | moderate | moderate | moderate | low | low | moderate | moderate |
| Zhong, 2019 | moderate | moderate | moderate | moderate | moderate | moderate | moderate | moderate |
| Dehghan, 2020 | moderate | serious | moderate | moderate | moderate | no information | moderate | serious |
| Drouin-Chartier, 2020 | moderate | moderate | low | low | low | low | moderate | moderate |
| Tong, 2020 | moderate | low | moderate | moderate | no information | low | low | moderate |
| Xia, 2020 | moderate | low | serious | moderate | low | low | low | serious |

**ESM** **Table 3. Dose-response meta-analysis of the association between egg intake and cardiovascular outcomes restricted to prospective cohort studies reporting complete data on number of participants/ person-years and cases.**

|  |  | **Eggs/week, RR (95% CI)** | | | | | | | | | |  |  |  |
| --- | --- | --- | --- | --- | --- | --- | --- | --- | --- | --- | --- | --- | --- | --- |
| **Outcome** | **datasets (studies)** | **0** | **1** | **2** | **3** | **4** | **5** | **6** | **7** | **8** | **9** | ***I^2^*** | ***P_heterog_*** | ***P_nonlinearity_*** |
| CHD | 15 (10) | 1 (ref.) | 0.97 (0.94; 1.01) | 0.95 (0.89; 1.01) | 0.94 (0.86; 1.02) | 0.94 (0.86; 1.03) | 0.95 (0.86; 1.05) | 0.97 (0.87; 1.07) | 0.98 (0.88; 1.09) | 1 (0.88; 1.12) | 1.01 (0.89; 1.16) | 87.35 | <0.001 | 0.051 |
| CHD mortality | 7 (5) | 1 (ref.) | 0.99 (0.95; 1.03) | 0.98 (0.91; 1.06) | 0.97 (0.89; 1.06) | 0.96 (0.87; 1.05) | 0.94 (0.86; 1.02) | 0.92 (0.85; 1.01) | 0.91 (0.82; 1) | 0.89 (0.8; 1) | 0.88 (0.77; 1) | 0 | 0.898 | 0.759 |
| CHD incidence/mortality | 20 (12) | 1 (ref.) | 0.97 (0.95; 1.01) | 0.95 (0.9; 1.01) | 0.94 (0.87; 1.02) | 0.94 (0.87; 1.03) | 0.95 (0.87; 1.04) | 0.97 (0.88; 1.06) | 0.98 (0.89; 1.09) | 0.99 (0.89; 1.11) | 1.01 (0.89; 1.14) | 83.29 | <0.001 | 0.058 |
| Stroke | 13 (9) | 1 (ref.) | 0.99 (0.97; 1.02) | 0.99 (0.94; 1.04) | 0.98 (0.92; 1.05) | 0.98 (0.92; 1.06) | 0.99 (0.92; 1.06) | 0.99 (0.93; 1.06) | 1 (0.94; 1.07) | 1.01 (0.94; 1.07) | 1.01 (0.95; 1.08) | 31.89 | 0.065 | 0.436 |
| Stroke mortality | 7 (5) | 1 (ref.) | 1.02 (0.95; 1.1) | 1.03 (0.91; 1.17) | 1.03 (0.89; 1.18) | 0.99 (0.89; 1.1) | 0.95 (0.84; 1.08) | 0.91 (0.75; 1.1) | 0.87 (0.67; 1.14) | 0.84 (0.59; 1.19) | 0.8 (0.52; 1.24) | 44.73 | 0.041 | 0.426 |
| Stroke incidence/mortality | 20 (14) | 1 (ref.) | 1 (0.98; 1.03) | 1 (0.95; 1.05) | 1 (0.94; 1.06) | 0.99 (0.93; 1.05) | 0.98 (0.93; 1.04) | 0.97 (0.93; 1.02) | 0.96 (0.91; 1.02) | 0.96 (0.89; 1.02) | 0.95 (0.87; 1.03) | 40.16 | 0.006 | 0.665 |
| CVD | 11 (8) | 1 (ref.) | 0.97 (0.94; 1) | 0.94 (0.89; 1.01) | 0.93 (0.87; 1) | 0.93 (0.87; 0.99) | 0.93 (0.88; 0.99) | 0.94 (0.89; 0.99) | 0.94 (0.89; 1) | 0.95 (0.89; 1.01) | 0.95 (0.88; 1.03) | 77.42 | <0.001 | 0.172 |
| CVD mortality | 7 (6) | 1 (ref.) | 0.96 (0.93; 0.99) | 0.93 (0.88; 0.98) | 0.92 (0.87; 0.98) | 0.92 (0.87; 0.98) | 0.93 (0.87; 0.99) | 0.93 (0.86; 1.01) | 0.93 (0.85; 1.03) | 0.94 (0.84; 1.05) | 0.94 (0.83; 1.07) | 43.68 | 0.046 | 0.043 |
| CVD incidence/mortality | 15 (12) | 1 (ref.) | 0.97 (0.94; 1) | 0.94 (0.9; 0.99) | 0.93 (0.88; 0.99) | 0.93 (0.88; 0.98) | 0.94 (0.9; 0.98) | 0.95 (0.91; 0.99) | 0.95 (0.91; 1) | 0.96 (0.91; 1.02) | 0.97 (0.9; 1.04) | 71.2 | <0.001 | 0.069 |
| Stroke, ischemic | 8 (6) | 1 (ref.) | 0.97 (0.95; 0.99) | 0.94 (0.9; 0.99) | 0.92 (0.87; 0.98) | 0.92 (0.87; 0.97) | 0.93 (0.89; 0.97) | 0.94 (0.91; 0.97) | 0.95 (0.91; 0.99) | 0.96 (0.9; 1.02) | 0.97 (0.89; 1.06) | 44.69 | 0.032 | 0.106 |
| Stroke, hemorrhagic | 7 (5) | 1 (ref.) | 0.94 (0.92; 0.96) | 0.88 (0.84; 0.93) | 0.84 (0.79; 0.9) | 0.82 (0.77; 0.88) | 0.82 (0.77; 0.87) | 0.82 (0.77; 0.87) | 0.81 (0.76; 0.87) | 0.81 (0.76; 0.87) | 0.81 (0.75; 0.88) | 0 | 0.893 | 0.001 |
| Heart failure | 6 (4) | 1 (ref.) | 1.01 (0.96; 1.05) | 1.01 (0.93; 1.1) | 1.02 (0.91; 1.15) | 1.04 (0.92; 1.19) | 1.07 (0.95; 1.21) | 1.11 (0.99; 1.25) | 1.15 (1.02; 1.3) | 1.19 (1.04; 1.36) | 1.23 (1.06; 1.44) | 37.05 | 0.103 | 0.409 |
